# 

**Published:** 1886-03

**Authors:** 


10 cts. a copy. MARCH, 1886. $1.00 per year.

### HALL'S JOURNAL of HEALTH

ESTABLISHED 1854

OFFICE, 75 & 77 BARCLAY STREET, NEW YORK.

Entered as Second-class Matter at the Post-Office, New York.

